# A simplified model for the combined wicking and evaporation of a NaCl solution in limestone

**DOI:** 10.1617/s11527-018-1187-y

**Published:** 2018-05-09

**Authors:** L. Pel, R. Pishkari, M. Casti

**Affiliations:** 1 Department of Applied Physics, Eindhoven University of Technology, Den Dolech 2, 5600 MB Eindhoven, The Netherlands; 2 Università degli Studi di Cagliari, Cagliari, Italy

**Keywords:** Diffusion, Advection, Wick action, NMR

## Abstract

Salt weathering is one of the major causes of the damage both in cultural heritage as well as in civil engineering constructions. A special case develops when there is a continuous wicking of a salt solution into a material in combination with evaporation of the moisture at its surface. In this study we are interested in the case where the absorption rate is much higher than the evaporation and as a result a salt concentration will build up at the drying surface resulting in crystallization. To this end we propose a simplified model to describe this mechanism. In order to check the model the NaCl concentration profiles were measured non-destructively by Nuclear Magnetic Resonance during a combined wicking and evaporation experiment with limestone. A good correlation was found between the model and the measured NaCl concentration profiles.

## Introduction

Salt weathering is one of the major causes of the destruction of our cultural heritage. Ions such as chlorides, sulphates and nitrates are well known to be the cause; the damage is by the crystallization of these soluble salts within the pores. These salts can only be transported within liquid. Therefore the liquid transport processes are dominant in determining where the accumulation will take place and therefore where salt crystallization and possible damage can occur. Often there is a continuous wicking of a salt solution. This can be encountered if the construction is in contact with groundwater or as often seen in marine environments when it is in contact with seawater. In these circumstances ions will be advected along with the capillary moisture flow into the material (see. e.g., [1, 2]).

When there is continuous wicking of a solution into a material in combination with evaporation of the moisture at the surface of the material an equilibrium will develop between wicking of the salt solution and the evaporation flux at the drying surface. Based on the sharp front model Hall [3] has shown, in the case the influence of gravity can be neglected, the evaporation front will lie either near one of the boundaries, i.e., the absorption or the drying interface. In this study we are interested in the case when the absorption rate is much higher than the evaporation. Hence, the material is saturated all the time and the drying front will be at the evaporation surface. This reflects the majority of the cases found in cultural heritage and civil engineering. A well-known example is the housing along the canals in the historic city of Venice where this process gives rise to continuous salt damage. This situation is not only encountered in cultural heritage but also for instance in concrete elements in a tunnel. Whereas cases in a marine environment can be identified quite easily, one should also consider monuments in contact with natural groundwater. Ground water contains dissolved salts coming from natural sources such salts in soil, rock, and organic material, or human activities, e.g., agricultural chemicals etc. Although the salt concentration might be very low, salts can still accumulate over many years or decades at the drying surface and give rise to crystallization damage. It has been recognized that this combined wicking and evaporation is an important process and as such lab tests have been used to look at the damage potential of various types of salts [4, 5].

One of the very first studies about combined wicking and evaporation action was carried out by Lewin [6]. Although his model gives some insight, it is an oversimplification. A more detailed study in one dimension has been done by Puyate et al. [7]. However in their model there is no explicit dependence on the drying rate. Most studies have focused on the boundary conditions trying to determine the effect of salt on the evaporation in order to get a better understanding of especially the type of the crystallization, i.e., efflorescence and subflorescence [8–10].

In this study the focus was on the NaCl transport in a porous material, i.e., limestone, along with the moisture flow and in particular on the build-up of the concentration near the drying surface. To this end we will first discuss a simplified model to describe the concentration build up. The model was checked by performing non-destructive measurement of the concentration build up using Nuclear Magnetic Resonance (NMR). This setup will be briefly discussed in the next sections, as well as the influence of the one-dimensional resolution of the NMR on the measured concentration profiles. Finally the results of the measurements will be presented and discussed.

## Theory

**Fig. 1:**
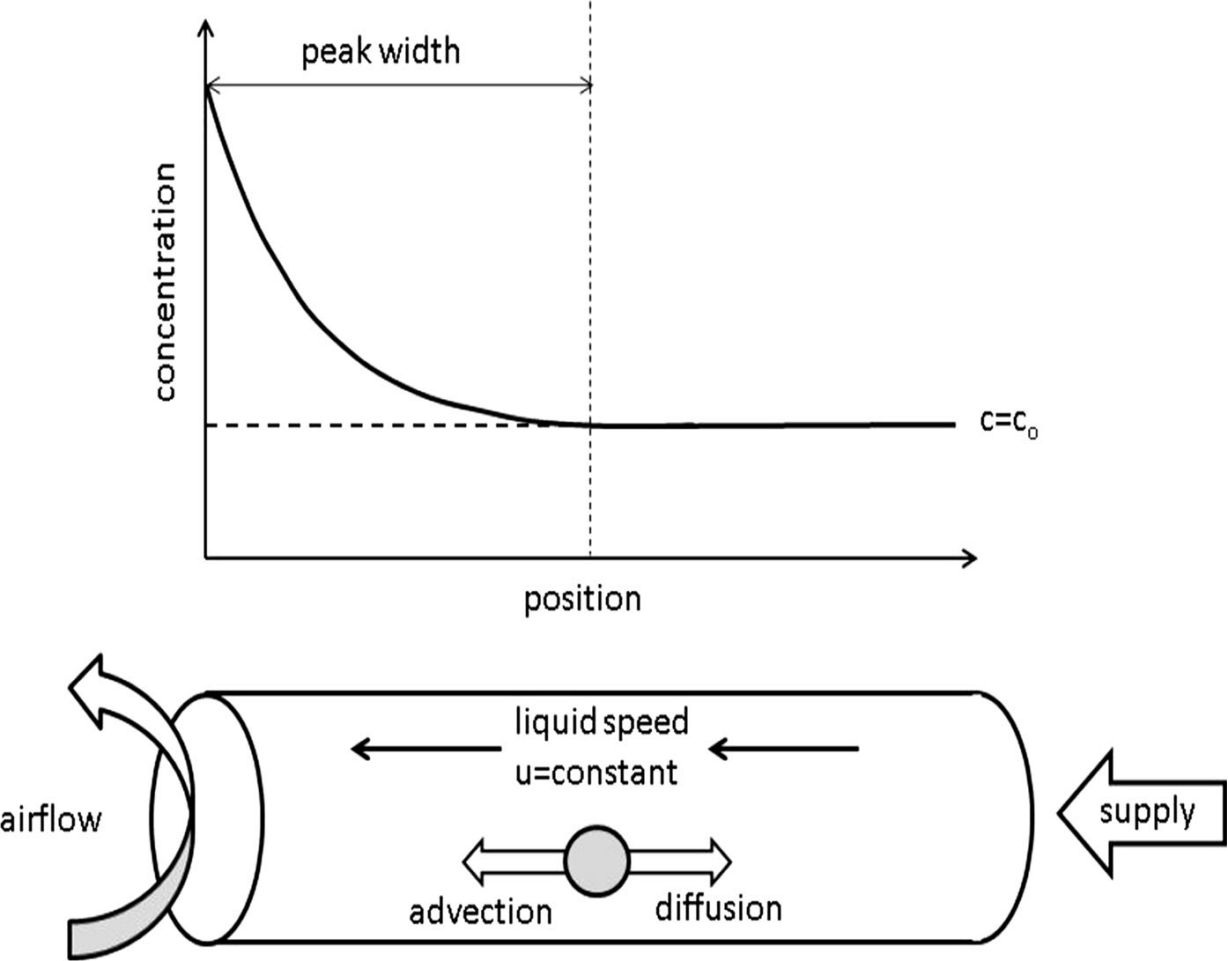
A schematic representation of a combined wicking and evaporation process: one side of an object is wicking a salt solution, whereas at the same time other side is drying. As a result there will be a continuous flow of ions towards the drying surface resulting in a concentration peak built up

In Fig. 1 a schematic representation of a combined wicking and evaporation experiment is given. In case the sample stays saturated, as is the case in the one dimensional experiments performed in this study and there is no crystallization, the ion transport can be described by an advection–diffusion equation (see, e.g., [11]):

1 $$\frac{\partial c}{\partial t} = \frac{\partial }{\partial x}\left( {D^{*} \frac{\partial c}{\partial x} + uc} \right)$$

where *c* is the ion concentration, D^*^ the macroscopic diffusivity of the ions within the porous material and *u* the macroscopic velocity of the liquid in the porous material, i.e., the Darcy speed.

The macroscopic diffusivity of the ions within a porous material is related to the microscopic diffusivity of the ions through the pores by the tortuosity T^*^, i.e.;

2 $$D^{*} = \frac{D}{{T^{*} }}$$

where T^*^ > 1 is a correction factor for the increase in path length due to the tortuous nature of pores.

Therefore the first part of the right hand side of Eq. 1 is describing the ion flux due to diffusion, whereas the second part describes the advection of the ions along with the liquid flow. Here advection will give rise to accumulation, whereas diffusion will give rise to levelling off of the concentration. These two competing processes can be characterized with a dimensionless number, i.e., the Peclet number. In this case, based on Eq. 1 it can be defined as [12]:

3 $$P_{e} = \frac{uL}{{D^{*} }}$$

where L is a so-called characteristic length scale, which in this case was chosen as the length of the sample. In the case *P*_e_ > 1, advection will be dominant and there will be a concentration gradient and as a result the salts will accumulate near the surface. Whereas in the case of *P*_e_ < 1, diffusion is dominant and we expect a homogenous distribution of salt. This Pe-number was also found to be very useful for giving an indication of the effect of poulticing [13]. In this paper we only address the case of *P*_e_ > 1, where ions will be advected towards the surface and there will be a concentration build up near the interface giving rise to crystallization.

As the sample is completely saturated the liquid speed, *u*, will be constant throughout the sample. The liquid speed will be completely determined by the drying condition at the top, i.e., the liquid flux *q*_*l*_ at the surface is given by:

4 $$q_{l} = \beta \left( {h_{\text{air}} - h_{\text{m}} \left( {c,\theta } \right)} \right)$$

where *β* is the mass transfer coefficient which is dependent on many parameters such as the air velocity, porosity and surface roughness, *h*_a_ the relative humidity of the air and *h*_m_ the relative humidity of the materials at the interface, which is a function of both the moisture content *θ* and the salt concentration. As the salt concentration is slowly changing near the drying surface the liquid speed will change slowly in time even if the drying airflow is kept constant. Therefore the salt concentration will have a large influence on the evaporation [14, 15].

As long as there is no crystallization at the drying surface the boundary condition for the ion transport is determined by the no flux condition, i.e.;

5 $$D^{*} \frac{\partial c}{\partial x} + uc = 0$$

At the wicking surface where the salt solution is absorbed the boundary condition is given by a constant flux, i.e.;

6 $$q_{\text{salt}} = uc_{o}$$

where *c*_*o*_ is the concentration of the liquid being absorbed at the wicking surface If we now assume that in the first-order approximation the liquid flow throughout the sample and the concentration at the inflow are constant, we can solve the partial differential Eq. 5, to as arrive at a first-order approximation of the ion concentration profiles in the experiment:

7 $$c\left( {x,t} \right) = \alpha \left( t \right)e^{{\frac{{ - D^{*} }}{u}x}} + c_{o}$$

where *α*(*t*) is a constant, which is a function of the time reflecting the increase of the concentration at the boundary. If we assume that the liquid velocity is constant, the increase of total ion content will be linear in time due to the regular advection of ions. Therefore by integrating Eq. 7 over the sample length *l* (here it is assumed that l > 4D^*^/u) we can get an expression for *α*(*t*), i.e.;

8 $$\alpha \left( t \right) = c_{o} \frac{{u^{2} }}{{D^{*} }}t + c_{o} \frac{u}{{D^{*} }}l$$

Hence it can be seen that as long as the liquid speed is constant, the concentration rise at the surface will increase linearly with time.

The solution of the concentration as function of time and space indicates that the ion concentration profiles can be described by a simple exponential decay which is dependent on the ratio between the diffusion and the liquid velocity. This dependence reflects the competition between advection and diffusion. One can define a peak width over which the concentration drops to about 2% of the original peak height α(t)-c_o_ as (see also Eq. 7):

9 $$\Delta = 4\frac{{D^{*} }}{u}$$

Hence as the ion diffusivity is constant, the peak width is fully determined by the liquid velocity and as the liquid velocity increases the peak width near the surface will decrease.

As soon as the ion concentration has reached the threshold level for crystallization, this mechanism will start and in this case the ion transport can be described by adding a sink term to Eq. 1 to account for crystallization, i.e.;

10 $$\frac{\partial c}{\partial t} = \frac{\partial }{\partial x}\left( {D^{*} \frac{\partial c}{\partial x} + uc} \right) + \gamma \left( {c - c^{*} } \right)$$

where c^*^ is the threshold concentration at which the crystallization starts and γ is a crystallization rate. In this case the crystallization term does not only indicate whether the crystallization will take place either inside near or outside at the surface of the sample.

## NMR

### NMR setup

**Fig. 2:**
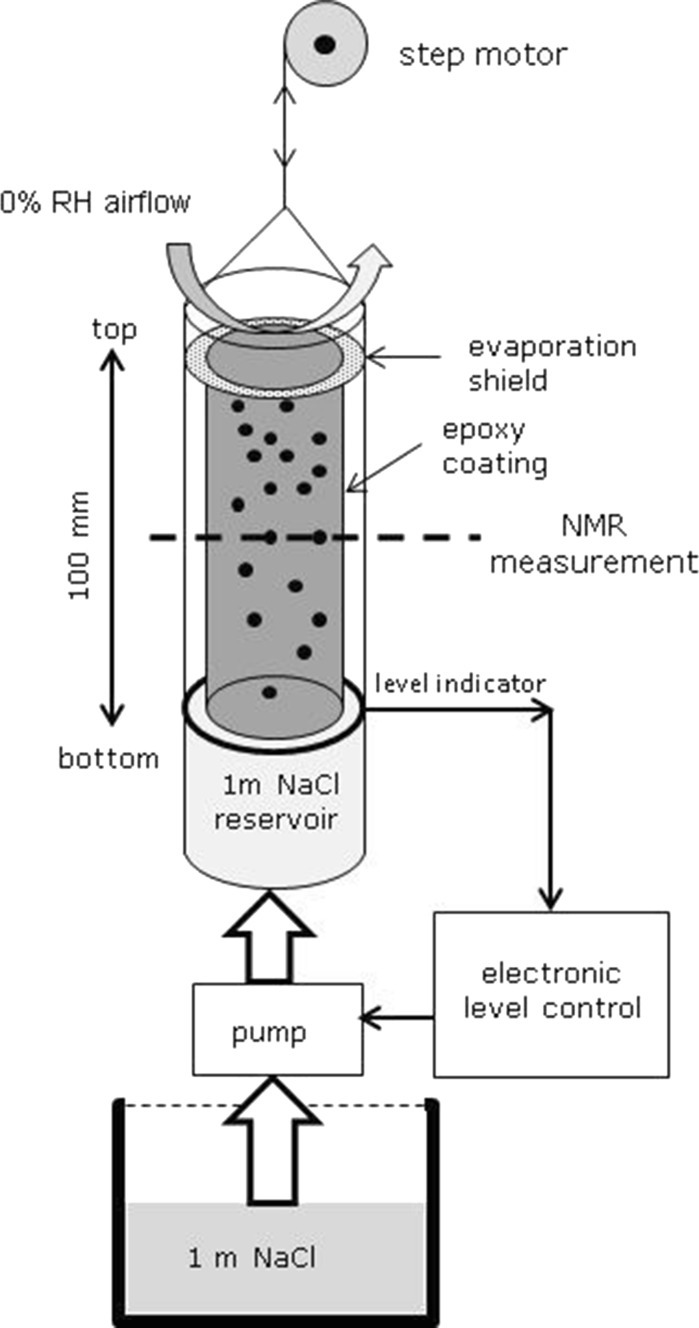
A schematic overview of the setup used for measuring the NaCl concentration profiles during wicking in combination with evaporation. The cylindrical samples are 20 mm in diameter and have a length of 100 mm. Using an electronic level indicator and a pump the level in the sample reservoir is kept constant. Using a step motor the sample is moved through the NMR, where at each position both the moisture content and the NaCl concentration are measured. The measurement time of one moisture and ion profile is in the order of 3 h

Nuclear Magnetic Resonance (NMR) was used for the measurement described in this study. This methods allows a non-destructively and quantitative measurement of both the moisture and NaCl-concentration profiles in an experiment (for more details on the setup see, e.g., [16, 17]). The experimental setup is provided in Fig. 2. In the experiments cylindrical samples of 20 mm diameter and 100 mm length were used. The side of the sample was sealed off with an epoxy coating, insuring the experiment can be considered as one-dimensional. The bottom of the sample is in contact with a reservoir filled with 1 m NaCl (mole/kg) solution. The level of this reservoir is maintained to be constant by a combination of an electric level indicator and a pump in contact with a large reservoir of 1 m NaCl solution. Simultaneously, there is a constant airflow (0% RH) over the top of sample in order to dry it and induce a constant moisture flow through the sample. In order to make sure the airflow has no influence on the reservoir concentration, i.e., evaporation of the reservoir, there is a seal separating the drying and wicking part of the experiment at the very top.

The sample is moved through the NMR with the help of a stepper motor. The moisture is first measured at the center of the NMR, after which the NMR machine is switched over to sodium and its content is then measured in order to determine the NaCl concentration. Subsequently the sample is moved to a new position and the procedure is repeated until a complete moisture and NaCl concentration profile is measured. Measuring the moisture content takes in the order of 40 s, where as it takes about 120 s to measure the Na concentration. With the NMR relaxation parameters used in the NMR experiment no signal is obtained from salt crystals and only free moving ions are measured. The measurement time of a complete profile takes is in the order of 3 h, whereas the total experiment took 50 days; hence variations of the profile during a measurement can be discarded.

### One-dimensional resolution

**Fig. 3:**
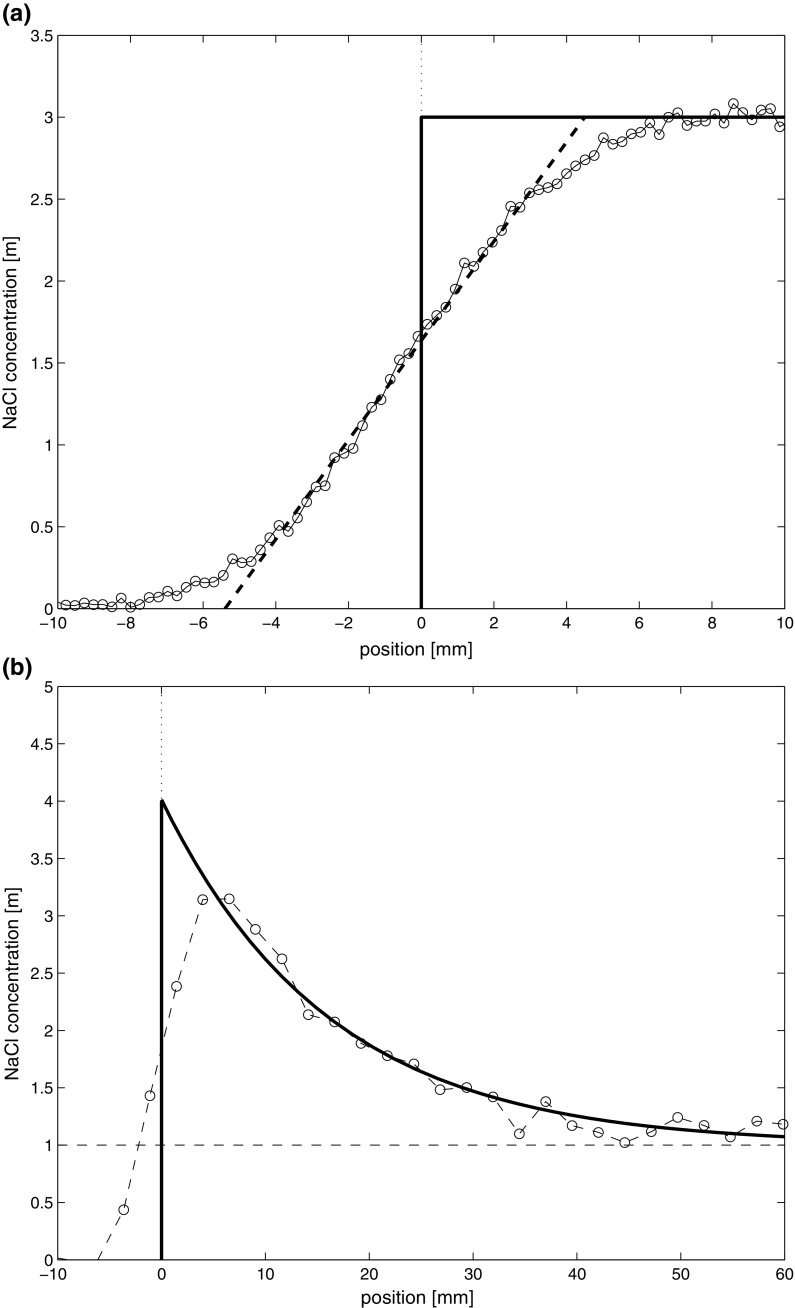
Two examples of the effect of the one dimensional resolution of the NMR on the measured NaCl profiles, i.e.; **a** the end of the sample is homogeneously saturated with 3 m, **b** a sample with an exponential decaying NaCl content profile. The original data is indicated by a solid line, whereas the measured concentration profiles by NMR are given by the interconnected circles

The NMR measurements are performed with a given one dimensional NMR resolution, reflecting the length scale over which the measurements are averaged, i.e., every measurement point is the average over a certain length, analogue to cutting the sample up into slices of a certain thickness. At the same time, the step motor allows to measure a point every 0.5 mm as the resolution of the step motor is much higher. As a result of the one dimensional NMR resolution, profiles will be smoothened. An example is shown in Fig. 3 where the results for measurements of the Na concentration of two samples are given. One sample is completely saturated where measuring the end reflects a step function (Fig. 3a) and the another sample with a salt profile reflecting an exponential decay (Fig. 3b). As can be seen for the step function the front is replaced by a linear increase, from 3 mm outside of the sample to 3 mm inside, which reflects the one dimensional resolution of 6 mm of the NMR setup. This one dimensional resolution is a function of the magnetic field gradient used in the NMR experiment. For the exponential decay we again see a linear increase, but in this case we see that due to the one-dimensional resolution the profile is smoothened off, which leads to underestimation of the maximum concentration in the sample.

## Measurements

### Material

The material used in these experiments is a biomicritic limestone from Sardinia. This limestone is very widespread in the Mediterranean basin and has been much used in buildings and monuments because it can be easily carved. These materials are characterized by a high porosity of 34% and poor mechanical properties so it weathers easily [18–20]. The average pore size has a maximum distribution at 1.6 µm (as measured by differential scanning calorimetry and Mercury intrusion porosimetry methods). The specimens were initially oven dried and then vacuum saturated with water before the experiments were carried out.

### Diffusion

**Fig. 4:**
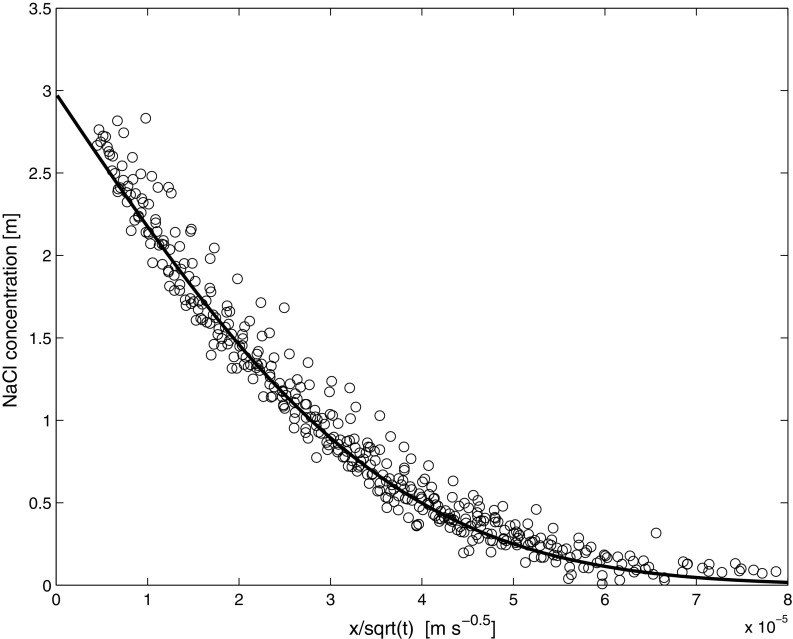
The measured NaCl concentration profiles after applying the Boltzmann–Matano transformation during diffusion of a 3 m NaCl (mole/kg) solution. The solid line is a fit through the data of the analytic solution, i.e., erf-function

Before the combined wicking/drying experiments was initiated the diffusion of a 3 m NaCl (mole/kg) solution in a sample was measured. The NaCl concentration profiles during diffusion were measured for 72 h. The measured ion concentration profiles after applying the Boltzmann–Matano transformation [21] are given in Fig. 4. As can be seen, after this transformation all profiles fall on one master curve indicating a pure diffusion process. In order to determine the diffusion coefficient we have fitted the analytic solution of the diffusion equation to the data, i.e., an error function (see for, e.g., [22]). Here we find D^*^ = 0.48 10^−9^ m^2^ s^−1^. As the bulk diffusion coefficient of NaCl in water is on average in the order of 1.5 10^−9^ m^2^ s^−1^ in the range from 1 to 6 m [23] we find that T^*^ = 3, which is in the order of values as reported in literature for many coarse porous building materials and stones [24, 25].

### Combined wicking and evaporation

**Fig. 5:**
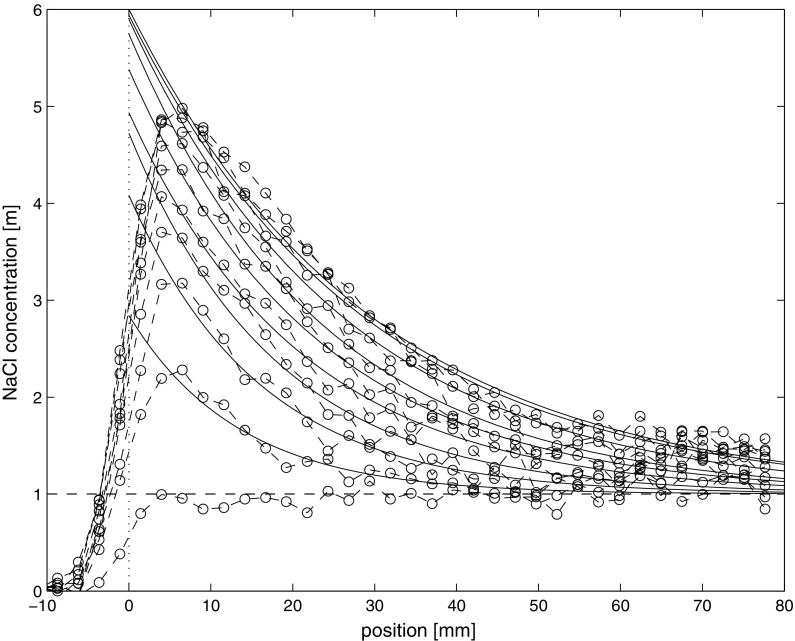
The measured NaCl concentration profiles by NMR during the combined wicking and evaporation of 1 m NaCl (mole/kg) solution at x = 100 mm and drying at x = 0 mm. The profiles are given for every 5 days from the moment on the sample is homogenous at 1 m for a total of 50 days. The interconnect circles with dashed lines indicate the raw measurement profiles. The solid lines are individual fits of the analytic model to the measured profiles. An air flow of 0% RH was blown over the top of the sample

The results of the combined wick action and evaporation experiment are given in Fig. 5. Although the NaCl concentration profiles were measured every 3 h, the figure shows the profiles for every 5 days for the first 50 days of the experiment, to reflect the slow process. As can be seen from these NaCl profiles the concentration near the surface slowly increases, whereas the concentration of the back remains constant at 1 m (mole/kg) reflecting the constant concentration of the inflow and of the reservoir. On the other hand we do not observe the concentration rising to the saturation concentration of 6.1 m as was expected for NaCl. Indeed if we inspect the profiles closer, the NaCl concentration rises from almost 0 at the position of 3 mm outside of the sample, i.e., in the air, to a maximum of 4 m at the position of 3 mm inside the sample. This gradual increase near the surface is due to 1D-resolution of the NMR, which is averaging each measurement point over 6 mm as already explained previously. In order to correct for this problem we have chosen to fit the simplified analytic model as given by Eq. 7 to each individual measured NaCl concentration profiles. These fitted profiles are provided by the solid lines in Fig. 5. As can be seen this exponential decay generated by this simplified model accurately describes the measured profiles quite well.

**Fig. 6:**
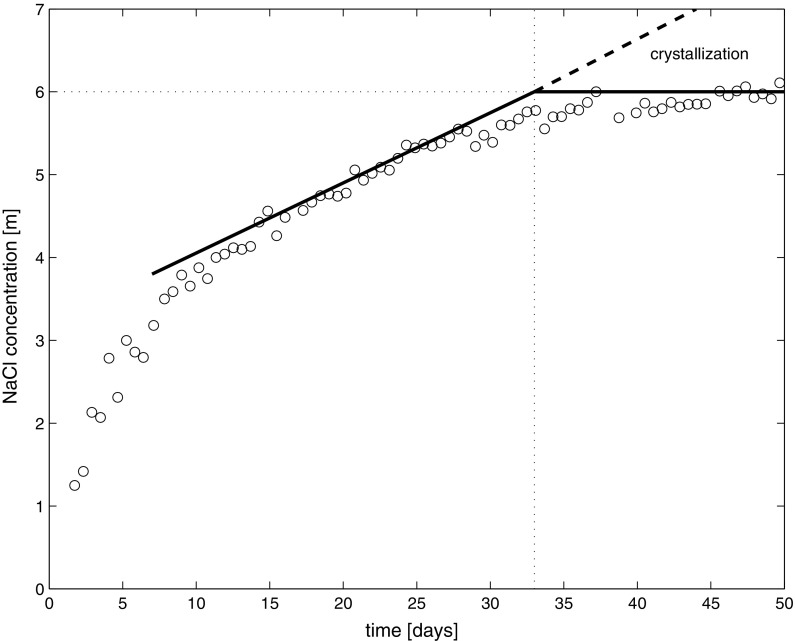
The NaCl concentration at the drying surface, of the sample i.e., x = 0 (see Fig. 5), as determined from fitting the individual measured NaCl concentration profiles during the experiment with the model

**Fig. 7:**
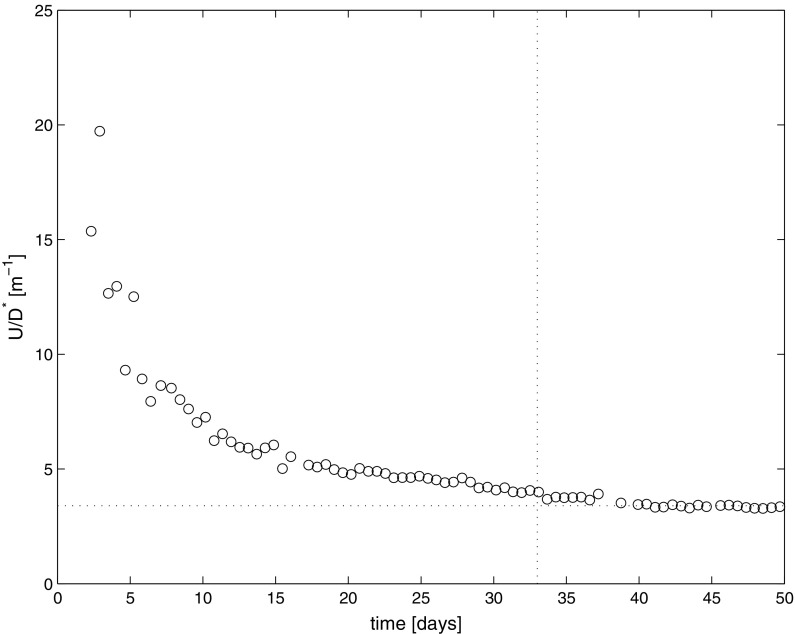
The ratio of liquid flow to diffusion within the sample as determined from fitting the individual measured NaCl concentration profiles during the experiment (see Fig. 5)

From the fitted profiles we can determine the NaCl concentration at the drying surface. The results for all measured concentration profiles are given in Fig. 6. Moreover in Fig. 7 we have plotted *u*/D^*^ as determined from the individual fits of the profiles. Since D^*^ is almost constant, within a first-order approximation this figure reflects the change in the liquid flow speed throughout our experiment. As can be seen during the first 5–10 days, as the concentration rises rapidly at the surface to 4 m, the liquid speed is dropping fast. As the sample has a fixed length of 0.1 m we see that the Pe-number is initially in the order of 20 and is decreasing to 3.5 towards the end of the experiment. This reflects the rapid change at the boundary as the NaCl concentration increases which will have a large influence on the sorption isotherm and therefore on the evaporation. After 10 days the liquid speed remains constant to the first-order. This is reflected in the almost linear increase of the concentration at the surface as indicated by the simplified model.

As can furthermore be seen from fitted profile as provided in Fig. 6, as soon as at the surface the concentration of the fitted profiles reaches 6 m crystallization starts and the concentration levels off. This indicates that in the current experiment no large supersaturation was measured, in contrast to experiments in glass capillaries where supersaturation ratios up to 1.6 were measured [26]. One could argue that the one-dimensional resolution is too low to measure the supersaturation in this particular experiment. However the buildup of such a supersaturation peak should also comply to the advection–diffusion model and hence to the fitted profiles. Moreover as indicated by Naillon et al. [27] as the crystallization initiates the supersaturation will be consumed very fast and indeed within the time resolution of this experiment no supersaturation was observed.

As can be seen as soon as the 6 m is reached and crystallization starts, the measured NaCl profiles does not significantly change. This indicates that crystallization must take place at the top of the sample. Indeed after the experiment was performed, a thick layer of salt was found on the top of the sample and no mechanical damage to the stone was observed, showing that the crystallization rate is high enough to compensate for the ion influx.

## Conclusion and discussion

The aim of this paper was to investigate the ion transport during combined wicking and evaporation; a situation which is often encountered in situ. It has been demonstrated by NMR that the NaCl concentration profiles can be measured non-destructively and quantitatively. The measured NaCl concentration profiles can be described by a simplified model, which only takes into account the liquid flow speed and the diffusivity of the ions in the material. This model reveals that although this combined wicking and evaporation is a slow process the concentration will continue to increase for many years, giving rise to salt crystallization and eventual damage. This combined process is dependent on the evaporation at the surface and therefore will strongly depend on the external condition during the yearly cycle in situ (see e.g. [28]).
